# Low-Intensity Focused Ultrasound Alleviates Spasticity and Increases Expression of the Neuronal K-Cl Cotransporter in the L4–L5 Sections of Rats Following Spinal Cord Injury

**DOI:** 10.3389/fncel.2022.882127

**Published:** 2022-05-12

**Authors:** Ye-Hui Liao, Mo-Xian Chen, Shao-Chun Chen, Kai-Xuan Luo, Bing Wang, Li-Juan Ao, Yao Liu

**Affiliations:** ^1^School of Rehabilitation, Kunming Medical University, Kunming, China; ^2^Department of Orthopaedics, Affiliated Hospital of Southwest Medical University, Luzhou, China

**Keywords:** low-intensity focused ultrasound, neurocircuits, spasticity, spinal cord injury, KCC_2_

## Abstract

Low-intensity focused ultrasound (LIFU) has been shown to provide effective activation of the spinal cord neurocircuits. The aim of this study was to investigate the effects of LIFU in order to alleviate spasticity following spinal cord injury (SCI) by activating the spinal neurocircuits and increasing the expression of the neuronal K-Cl cotransporter KCC_2_. Adult male Sprague Dawley (SD) rats (220–300 g) were randomly divided into a sham control group, a LIFU^−^ group, and a LIFU^+^ group. The mechanical threshold hold (g) was used to evaluate the behavioral characteristics of spasm. Electromyography (EMG) was used to assess activation of the spinal cord neurocircuits and muscle spontaneous contraction. Spasticity was assessed by frequency-dependent depression (FDD). The expression of KCC_2_ of the lumbar spinal cord was determined via western blot (WB) and immunofluorescence (IF) staining. The spinal cord neurocircuits were activated by LIFU simulation, which significantly reduced the mechanical threshold (g), FDD, and EMG recordings (s) after 4 weeks of treatment. WB and IF staining both demonstrated that the expression of KCC_2_ was reduced in the LIFU^−^ group (*P* < 0.05). After 4 weeks of LIFU stimulation, expression of KCC_2_ had significantly increased (*P* < 0.05) in the LIFU^+^ group compared with the LIFU^−^ group. Thus, we hypothesized that LIFU treatment can alleviate spasticity effectively and upregulate the expression of KCC_2_ in the L4–L5 section of SCI rats.

## Introduction

Spasticity (involuntary contractions of paralyzed muscles) is an important complication of patients suffering from spinal cord injury (SCI) or stroke, and seriously affects their quality of life (Holtz et al., 2017; Pan et al., 2022). Studies have shown that 65% of SCI patients suffer muscle hypertonia, hyperreflexia, and spasticity (Sköld et al., 1999; Holtz et al., 2017). The pathogenesis of spasticity following SCI is complex, while increasing excitability of the motor neurons below the spinal damage lever play important roles (Sheean, 2002). In a normal spinal cord, the balance of excitation and inhibition plays an important role in physiological motor responses. Following SCI, descending input, such as serotoninergic descending tracts from upper motor neurons, become damaged, which contributes to disruption of the excitation/inhibition balance (Brocard et al., 2016). Loss of the serotoninergic descending tracts, the inhibitory interneurons, such as Renshaw cells connected to the motor neurons, and suppressing excitation of the motor neurons, can reduce feedback from GABAergic neurons (Carr et al., 1999; Wootz et al., 2013). With a reduction in efficient inhibition by the interneurons, the motor units below the SCI show a prolonged depolarization, and even with a brief sensory stimulation (<20 ms) (Lin et al., 2007). All of the above-mentioned factors were found to lead to spasticity following SCI (Boulenguez and Vinay, 2009; Boulenguez et al., 2010). Thus, increasing activation of the spinal cord neurocircuits above the SCI section would be a feasible method for reducing the excitability of the spinal cord below the SCI, thus alleviating spasticity (Kupcova Skalnikova et al., 2013).

Therefore, as one of the serious complications of upper motor neuron syndrome (Sheean, 2002), spasticity has a complex pathogenesis. However, there is still no effective treatment. Drugs, surgery, and rehabilitation therapy are often used to improve the symptoms of spasmodic patients. Moreover, there are still some deficiencies in the above treatment methods for such spasmodic symptoms, including, skin lesions caused by medical treatments (Lannin et al., 2007), muscle weakness and infection at the injection site induced by botulinum toxin injection (Elbasiouny et al., 2010), and muscle weakness caused by baclofen (Kirshblum, 1999), etc.

KCC_2_ is an important K^+^-Cl^−^ transporter on the cell membrane of mature motor neurons, which can remove intracellular Cl^−^ to the extracellular zone, thus maintaining a low concentration of chloride ions ([Cl^−^]_i_) in motor neurons (Nilius and Droogmans, 2003). Lower [Cl^−^]_i_ plays an important role in maintaining gamma-aminobutyric acid (GABA) and glycine inhibition for motor neurons (Viemari et al., 2011; Mazzone et al., 2021). It has been reported that the expression of KCC_2_ was significantly downregulated on the motor neurons of the spinal cord below the injury level of the spinal cord (Boulenguez et al., 2010). The downregulation of KCC_2_ in the cell membranes of motor neurons leads to increased intracellular [Cl^−^]_i_, weakening the inhibitory effect of GABA and glycine to motor neurons, increasing the excitability of motor neurons, and finally leading to limb spasticity (Mazzone et al., 2021).

As a potential non-invasive neuromodulatory technology, the therapeutic effect of low-intensity focused ultrasound (LIFU) on central nervous system diseases has been a focus of research in recent years (Legon et al., 2018; Lipsman et al., 2018; Darrow, 2019). According to animal experiments, eye movements, pupil dilation, and animal paw and/or tail movements were observed when the central nervous system was stimulated by LIFU, and changes in electromyography (EMG) signals were also detected (Darrow, 2019). A recent study confirmed that LIFU exerted effective action on the deep brain region and increased the expression of C-fos positive cells (Hou et al., 2021). It has been reported that LIFU irradiation of specific areas of the cerebral cortex in primates and humans can significantly reduce the amplitude of cortical-evoked potential and enhance the ability of tactile discrimination (Legon et al., 2018). Both cellular and animal experiments have confirmed that LIFU not only affects the activation and depolarization of Ca^2+^ and Na^+^ ion channels on the cell membrane (Kubanek et al., 2016) but also upregulates the expression of brain-derived neurotrophic factor (BDNF) following central nerve injury (Yang et al., 2015; Ni et al., 2017; Blackmore et al., 2019). In a previous study, our team also confirmed that LIFU stimulation can activate the spinal cord neurocircuits (Liao et al., 2021a) and increase the expression of KCC_2_ of the spinal cords of neuropathic pain rats effectively (Liao et al., 2021b). However, whether LIFU can activate the spinal cord neurocircuits, upregulate the expression of KCC_2_, and then alleviate spasticity post-SCI spasm is still not completely clear. In this study, we used LIFU to stimulate the lumbar spinal cords of SCI rats, electrophysiology tests and behavior assessment to evaluate its therapeutic effects, and western blotting (WB) and immunofluorescence (IF) staining to examine the expression of KCC_2_ in the lumbar spinal cord in order to explore the possible mechanism of LIFU treatment.

## Materials and Methods

### Animals and Experimental Drugs

**Figure 1:**
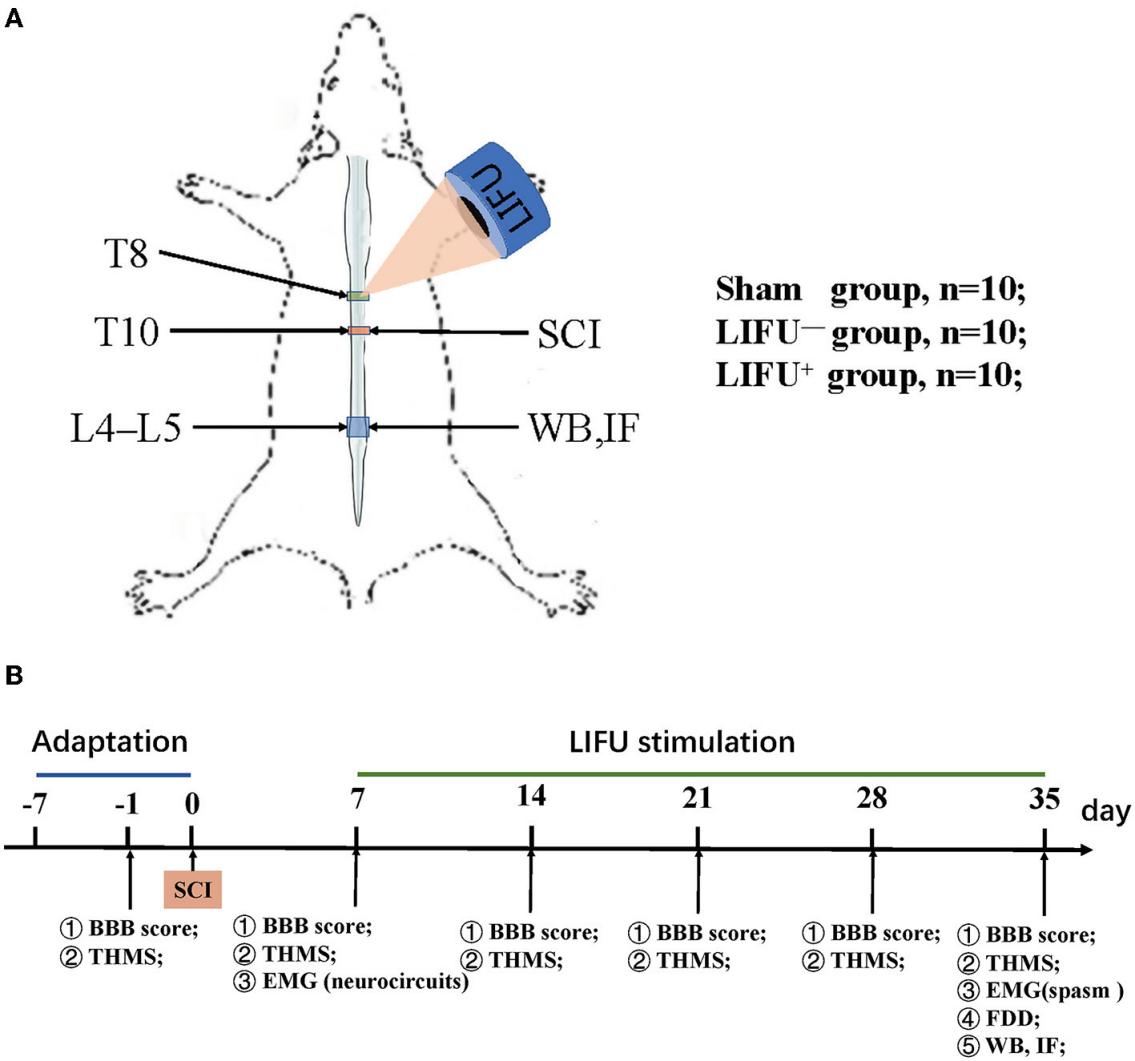
Experimental design of the study. **(A)** Modeling and ultrasound therapy. **(B)** Experimental design. The Basso, Beattie, and Bresnahan (BBB) score and threshold hold (g) of mechanical stimulation (THMS) were tested 1 d before the spinal cord injury (SCI) and weekly starting from 7 d post-injury (dpi); electromyography (EMG) of the neurocircuits and spasm were tested before/after low-intensity focused ultrasound (LIFU) treatment separately; frequency-dependent depression (FDD), western blot (WB), and immunofluorescence (IF) staining were tested following the end of LIFU treatment.

Adult male Sprague Dawley (SD) rats (200–280 g) were purchased from the Animal Experiment Center of Kunming Medical University, Yunnan Province, China. The animals were housed in a temperature-controlled facility with a day–night cycle of 12/12 h and free access to food and water. The study design is shown in Figure 1. A total of 30 animals were randomly divided into three groups: (i) a sham operation group (*n* = 10), in which the rats received all the surgical procedures except for SCI at T10; (ii) a LIFU^−^ group (*n* = 10), in which the rats received spinal cord injury (SCI) and LIFU treatment but with the ultrasonic amplifier always turned off; and (iii) a LIFU^+^ group (*n* = 10), where all the rats received SCI and LIFU treatment, as shown in Figure 1A. Neuromotor functional and behaviors assessment was performed at pre-SCI, 1 d pre-LIFU, and 1, 2, 3, 4 weeks post-LIFU; an EMG test for activation of the spinal cord neurocircuits was performed 1 d pre-LIFU; and EMG tests for spasm, H-reflex tests, WB, and IF staining were performed 4 weeks post-LIFU, as shown in Figure 1B.

### Surgical Procedures in Rats

The SCI surgical procedures were performed as described in previous reports (Brocard et al., 2016), and the spinal cord was transected at the T10 level. Briefly, rats were anesthetized with 2% isoflurane and fixed in the prone position. The hair on the back was shaved off and the skin at the T9–T11 vertebra was incised. The paravertebral muscles were dissected bluntly from T9 to T11. T10 laminectomy was performed carefully in order to expose the epidural and spinal cord, which were then cut transversally with sharp blades, followed by suturing of the paravertebral muscles and skin, layer by layer. The incision was disinfected once again, 0.9% NaCl was injected intraperitoneally to prevent dehydration, and the animals were placed in a 37°C incubator until awakening from anesthesia. Penicillin (160,000 units, qd) was postoperatively injected intraperitoneally for 3 d. Manually assisted urination was performed daily until the autonomous reflex of urination was restored.

### LIFU Treatment Program

LIFU stimulation of the spinal cord was performed as described previously (Liao et al., 2021a,b). Briefly, before LIFU treatment, the hair around the ultrasonic irradiation site on the back of each rat was shaved off. The ultrasonic probe was fixed with a clapper in order to ensure that the ultrasound focus was located at the spinal cord. An ultrasonic coupling agent was applied between the transducer and the spinal skin in order to ensure that there was no bubble in the space. The LIFU parameters were as follows: sine pulse, frequency = 4 MHz, pulse repetition rate (PRF) = 0.8 kHz, radiation intensity (RI) = 0.65 MPa, and duty cycle (DC) = 50%. The ultrasound system included a signal generator (DG4202, RIGOL, China), a power amplifier (Dahan Radio Studio, China), and an ultrasonic probe (DOBO, China). LIFU treatment was started 1 week after SCI, once a day, 20 min each time, for a total of 4 weeks. Calibrated hydrophones (2010, Precision Acoustics Ltd, Dorchester, UK) were used to measure the acoustic field distribution and the acoustic intensity parameters.

### Behaviors Assessment of Spasticity

The threshold hold (g) of mechanical stimulation (THMS) of the tail to induce spasm spasticity, such as a muscle spasm or hyperreflexia (Corleto et al., 2015), was recorded as described in the literature (Plantier et al., 2019). Generally, after the rats familiarized themselves with the test environment for 15 min, a microsensor that could detect pressure was installed between the fingers and the tail of the rat. When muscle spasm was observed in the hind limb, as shown in Supplementary Video 1, the pressure on the tail was recorded. If no muscle twitch or spasm was observed when the pressure was ≥3,000 g, the pressure was recorded as 3,000 g. The measurements were repeated three times, with an interval of 5 min, and the mean value of the three measurements was calculated.

### Neuromotor Functional Assessment

The Basso, Beattie, and Bresnahan (BBB) locomotor scale (Basso et al., 1995) was used to evaluate neuromotor function before and after LIFU treatment. The BBB scores ranged from 0 to 21 points, which included joint movement, gait coordination, and paw placement. Slight paralysis (score of 14–21) shows sustained hind leg coordination, moderate paralysis (score of 8–13) shows the ability to land on the palm without bearing weight, and severe paralysis (score of 0–7) shows no movement or slight movement of one or two joints (Yu et al., 2021).

### Electrophysiological Tests and Analysis

Electrophysiological assessment of spasticity included H-reflex and EMG, which have been described in the literature (Corleto et al., 2015; Beverungen et al., 2020). The EMG test was performed as previously described (Liao et al., 2021a). Briefly, the EMG signal was recorded using a concentric circular electrode, which was inserted into the soleus (Sol) muscle. The reference electrode was inserted percutaneously into the tail. The EMG signal was amplified by an A-M system, filtered over the range 200 Hz−5 kHz, and analyzed by a computer running Signal 5 software (Signal, Cambridge Electronics Design Ltd., Cambridge, UK). For the activation of the spinal cord neurocircuits test, the EMG was recorded when the spinal cord was receiving LIFU stimulation. The EMG test for hind limb spasm was evoked by a mechanical stimulation of the tail (1,500 g for 1 s stimuli, repeated three times at 10 s intervals).

For the H-reflex test, the rats were mildly anesthetized. A pair of stimulating electrodes, which were used for evoking the H-reflex, were inserted percutaneously around the tibial nerve, and a pair of recording electrodes, which were used for recording the H- and M-waves, were inserted percutaneously into the interosseous muscle at the fifth phalange. The reference electrode was inserted percutaneously into the tail. The H-reflex was induced by an independent pulse (100 μs) produced by the A-M system. The stimulus intensity that caused the maximum H-reflex was used to evoke frequency-dependent depression (FDD). A successive stimulus (20 times) was performed, and the H/M ratio was calculated by taking the average of the final 15 stimuli. The changes in H-reflex at 0.5, 1, 5, and 10 Hz were calculated as a percentage of the response obtained at 0.2 Hz. FDD data were presented as the mean ± standard error on the mean (SEM). After the last electrophysiological test, the rats were sacrifice for WB and IF staining.

### Western Blot

WB was used to examine the protein expression of KCC_2_ in L4–L5 spinal cord. After the final electrophysiological examination, the rats were given an intraperitoneal injection of an overdose of anesthetics perfused with ice saline (0.9% NaCl). The L4–L5 spinal cord was extracted and stored at −80°C immediately before the protein test. The spinal tissues (0.1 g) were lysed with radioimmunoprecipitation assay (RIPA) buffer (1 ml, Solarbio Life Sciences, Beijing, China) and phenylmethylsulfonyl fluoride (PMSF) (10 μl, Solarbio). The protein concentration was determined using a BCA protein assay kit (Beyotime Biotechnology, Shanghai, China) after centrifugation at 12,000 rpm at 4°C for 15 min. Following electrophoresis and transfer to nitrocellulose, the membrane was blocked with 5% skim (fat-free) milk for 2 h, washed with a mixture of tris-buffered saline and Polysorbate 20 (TBST) three times for 15 min each time, following incubation with primary antibody (KCC_2_, 1:1,000, Cell Signaling, Danvers, MA, USA; β-Actin, 1:4,000, Santa Cruz Biotechnology, Dallas, TX, USA) at 4°C overnight. After washing with TBST three times, the membranes were incubated with a second antibody (peroxidase-conjugated AffiniPure goat anti-mouse/rabbit, 1:2,000, ZSGB-BIO, Beijing, China) at room temperature for 2 h. ImageJ software (https://imagej.nih.gov/ij) was used to quantify the optical density of the protein bands.

### Immunofluorescence Staining

Following an overdose of anesthetics, the rats were perfused with phosphate-buffered saline (PBS) and 4% paraformaldehyde. The L4–L5 spinal cord was extracted and fixed with 4% paraformaldehyde overnight at room temperature. The fixed tissue was dehydrated with 10, 20, and 30% sucrose, step by step, following embedding in Tissue-Tek OCT medium and slicing into 6-μm-thick sections. After blocking with 3% bovine serum and 0.3% Triton X-100 in PBS, the sections were incubated with primary antibodies overnight at 4°C, followed by incubation with secondary antibodies for 2 h at room temperature. The primary antibodies used were rabbit antibody to KCC_2_ (1:100) and NeuN (1:100) (all Cell Signaling, USA). The secondary antibodies, anti-rabbit IgG (H + L), F(ab′)2 fragment (Alexa Fluor® 488 Conjugate), and anti-mouse IgG (H + L), F(ab′)2 fragment (Alexa Fluor® 594 Conjugate) were used for fluorescence staining. A fluorescence microscope (Olympus Corporation, Tokyo, Japan) was used to obtain the fluorescence images. ImageJ software was used to quantify the fluorescence intensity.

### Statistical Analysis

All of the data are presented as the mean ± SEM. One-way analysis of variance (ANOVA) and least significant difference (LSD) were used for comparing the different groups. When the results showed a difference, Fisher's protected LSD tests were used for pairwise comparisons. SPSS 20.0 software (IBM, New York, NY, USA) and Prism 8 (GraphPad Software Inc., San Diego, CA, USA) were used for statistical analysis and histogram making. Two-tailed *P*-values < 0.05 were considered statistically significant.

## Results

### Activation of Spinal Cord Neurocircuits by LIFU Stimulation

**Figure 2:**
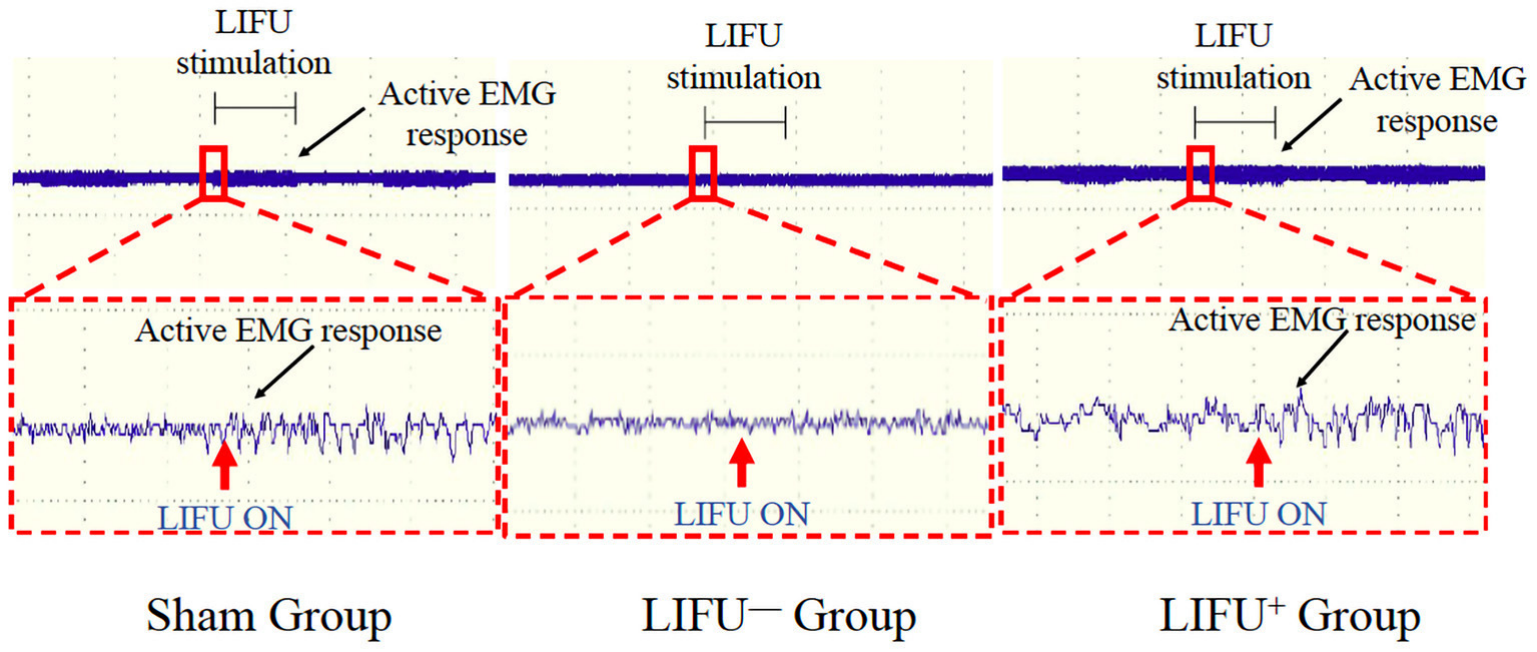
Activation of the spinal cord neurocircuits by low-intensity focused ultrasound (LIFU) stimulation. The red arrow shows the LIFU turning on point; the black arrow shows the active EMG response.

Activation of the spinal cord neurocircuits can lead to recruitment of the hind leg muscles and can be recorded by EMG. In this study, LIFU stimulation induced a significant recruitment of soleus muscle and was recorded by EMG, as shown in Figure 2. In the LIFU^+^ stimulation group, we could also clearly hear the EMG sound produced by the muscle recruitment with stimulation by LIFU, and without the EMG sound in the LIFU^−^ stimulation group, as shown in Supplementary Video 2.

### Effect of LIFU on Reducing Spasticity

**Figure 3:**
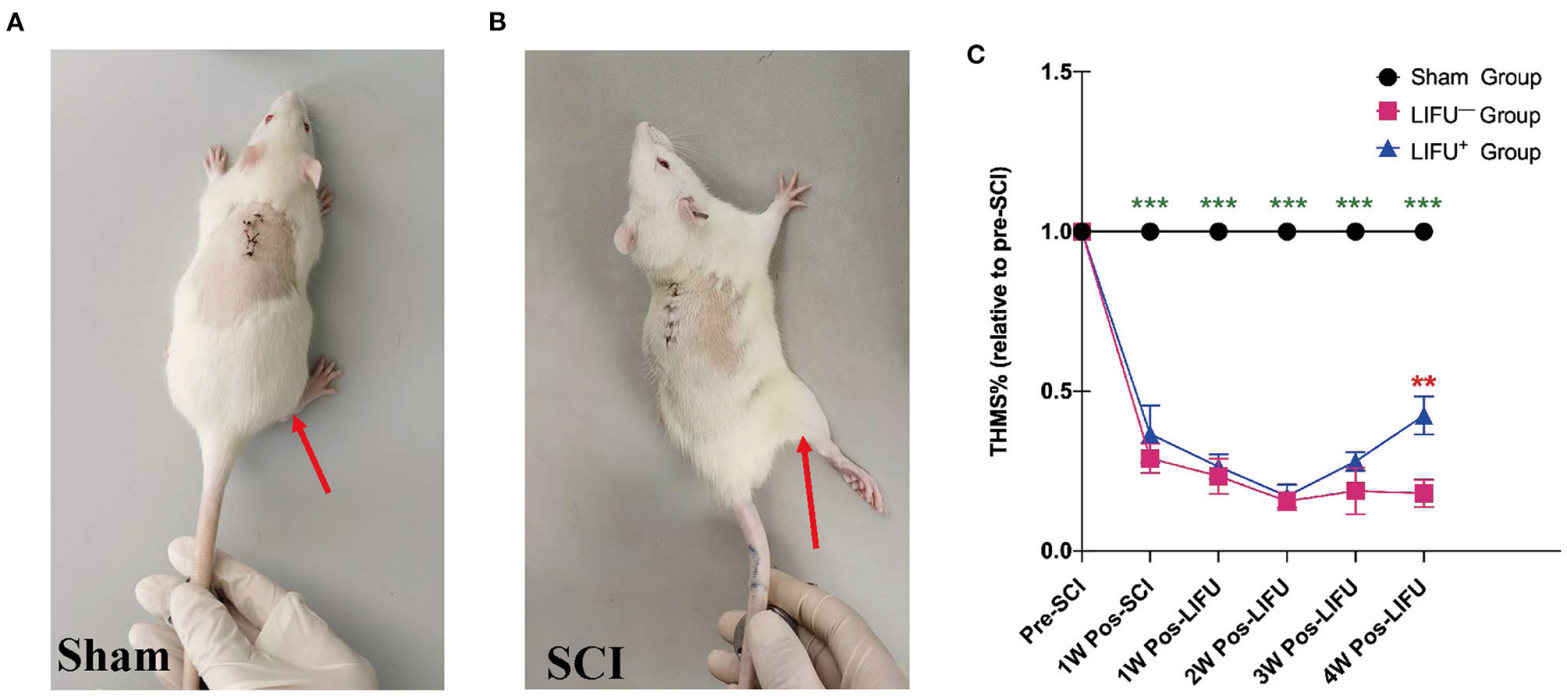
Threshold hold (g) of mechanical stimulation (THMS) test for the spasm. **(A)** A normal hind limb motor response to tail pinching was shown by the sham operation rats. **(B)** A typical hind limb motor response to tail pinching was shown by the spinal cord injury (SCI) rats. **(C)** This shows the THMS responses to tail pinching. ***P < 0.001, compared with the sham group; **P < 0.01, compared with the LIFU^−^ group; one-way analysis of variance (ANOVA) with a least significant difference (LSD) test; *n* = 10; mean ± standard error on the mean (SEM).

One week into the operation, the rats in the sham operation group showed no muscle twitch or hind limb spasm, as shown in Figure 3A, Supplementary Video 1; while in the SCI group, the rats showed significant muscle twitch and hind limb spasm following mechanical stimulation of the tail, as shown in Figure 3B, Supplementary Video 1. One week after SCI, the threshold of mechanical stimulation was significantly reduced in the LIFU^−^ and LIFU^+^ groups (*P* < 0.05) when compared with the sham group, but there was no difference between the LIFU^−^ and LIFU^+^ groups (*P* > 0.05). In the LIFU^+^ group, the mechanical stimulation threshold increased (*P* < 0.05) after 4 weeks of LIFU treatment when compared with the LIFU^−^ group, as shown in Figure 3C.

### Effect of LIFU on Increasing Neuromotor Function

Comparison of the BBB scores showed no significant differences (*P* > 0.05) among the three groups at the pre-SCI points. One week after SCI, the BBB score of the SCI group (LIFU^−^ and LIFU^+^) had reduced significantly (*P* < 0.05), but there was no significant difference between the LIFU^−^ and the LIFU^+^ group. After 4 weeks of LIFU stimulation, the BBB scores of the LIFU^+^ group increased compared with those of the LIFU^−^ group (*P* < 0.05), while still being below those of the sham group (*P* < 0.05), as listed in Table 1.

**Table 1:** BBB scores in sham, LIFU^−^ and LIFU^+^ group at different time pre- and pos- spinal cord injury(SCI) and low intensity focus ultrasound (LIFU) treatment.

	**Sham**	**LIFU^−^**	**LIFU^+^**
Pre-SCI	20.7 ± 0.15	20.5 ± 0.22	20.4 ± 0.26
1w-pos SCI	20.4 ± 0.27	0.8 ± 0.33***	1.1 ± 0.35***
1w-pos LIFU	20.2 ± 0.25	0.8 ± 0.32***	1.0 ± 0.33***
2w-pos LIFU	20.6 ± 0.22	1.0 ± 0.30***	1.3 ± 0.34***
3w-pos LIFU	20.4 ± 0.37	0.9 ± 0.25***	1.4 ± 0.34***
4w-pos LIFU	20.6 ± 0.22	1.3 ± 0.30***	2.8 ± 0.47^***##^

*** *P < 0.001, indicated significant difference when compared with Sham group*;

## *P < 0.01, indicated significant difference when compared with LIFU^–-^ group. n = 10, one-way ANOVA and LSD test*.

### LIFU Alleviated FDD and EMG

**Figure 4:**
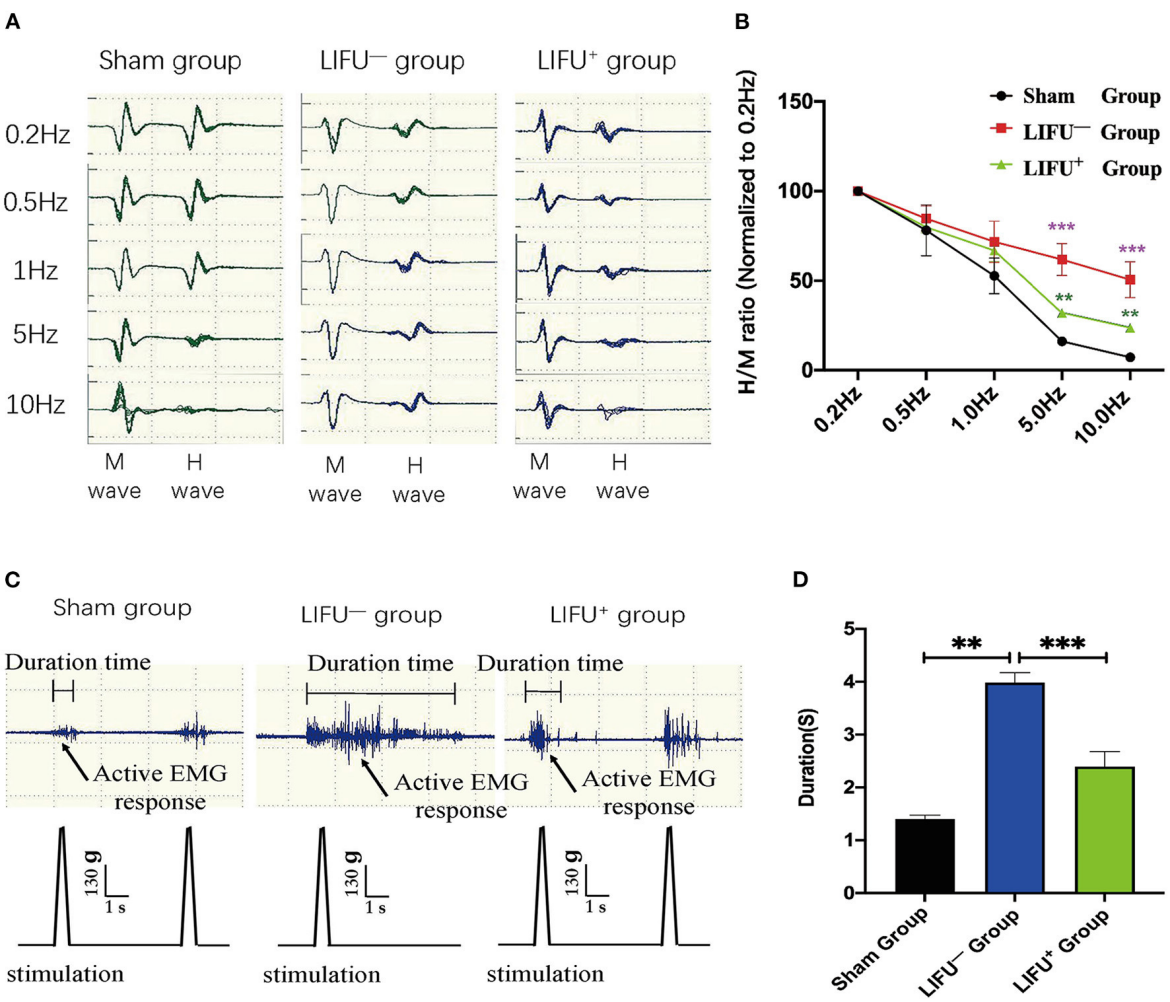
Change in frequency-dependent depression (FDD) and electromyograph (EMG) after 4 weeks of LIFU stimulation. **(A)** A typical H_max_/M_max_ recording over a series of 20 stimulations at 0.2, 0.5, 1, 5, and 10 Hz. The amplitude of the H-reflex decreased with increasing stimulus frequency. **(B)** FDD was normalized to intact (0.2 Hz) and showed significant differences among the different groups at 5 and 10 Hz stimuli; ***P < 0.001, compared with the sham group; **P < 0.05, compared with the LIFU^−^ group; one-way ANOVA with LSD test; *n* = 10, mean ± SEM. **(C)** a typical EMG recording; the black arrow shows the active EMG response following LIFU stimulation. **(D)** after 4 weeks of LIFU treatment; the durations of the EMGs were significantly different among the three groups; **P < 0.01 in the LIFU^−^ group compared with the sham group; ***P < 0.001 in the LIFU^+^ group compared with the LIFU^−^ group; one-way ANOVA with LSD test; *n* = 10, mean ± SEM.

FDD was shown to depress spasticity, as shown in Figure 4A. After 4 weeks of LIFU treatment, FDD showed no significant differences among the three groups at 0.5 and 1 Hz stimulation. After 5 and 10 Hz stimulation, however, the FDD of the LIFU^−^ group was significantly higher than that of the sham group (*P* < 0.05). Following LIFU stimulation, the FDD decreased, and that of the LIFU^+^ group was lower than that of the LIFU^−^ group (*P* < 0.05), as shown in Figure 4B. The EMG showed the response time of soleus muscle after stimulation of the tail, as shown in Figure 4C. The response time of the LIFU^−^ group was significantly longer than that of the sham group. Following LIFU stimulation, the response time of the LIFU^+^ group was shortened compared with that of the LIFU^−^ group, as shown in Figure 4D.

### Effects of LIFU on the Expression of Protein

**Figure 5:**
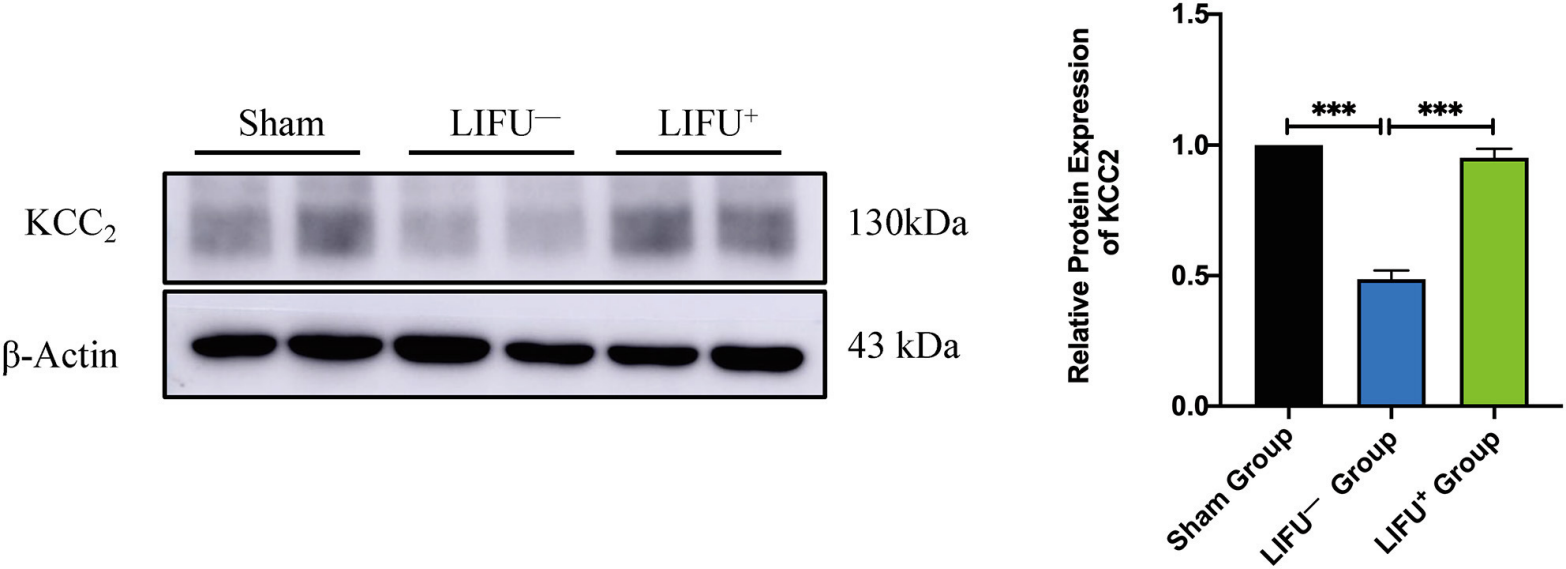
Protein expression of KCC_2_ in the sham group, LIFU^−^ group, and LIFU^+^ group after 4 weeks of LIFU treatment. Western blot (WB) showing the expression of KCC_2_; histogram showing the intensity of protein expression for the different groups. These result show that the intensity of KCC_2_ expression significantly decreased following SCI (***P < 0.001, compared with the sham group; one-way ANOVA with LSD test; *n* = 5, mean ± SEM); following LIFU treatment, the intensity of KCC_2_ expression significantly increased (***P < 0.001, compared with the LIFU^−^ group; one-way ANOVA with LSD test; *n* = 5, mean ± SEM).

Following SCI, the expression level of KCC_2_ was reduced (Boulenguez et al., 2010). Downregulation of KCC_2_ expression had been reported to be one of the important molecular mechanisms of limb spasm following SCI (Boulenguez et al., 2010). In this study, the expression levels of KCC_2_ in the lumbar L4–L5 spinal cord of the LIFU^−^ group were significantly downregulated compared with the sham group at 5 weeks following SCI. In the LIFU^+^ group, the expression of KCC_2_ was significantly upregulated (*P* < 0.05) compared with the LIFU^−^ group after 4 weeks of LIFU treatment (*P* < 0.05), as shown in Figure 5.

### Immunofluorescence

**Figure 6:**
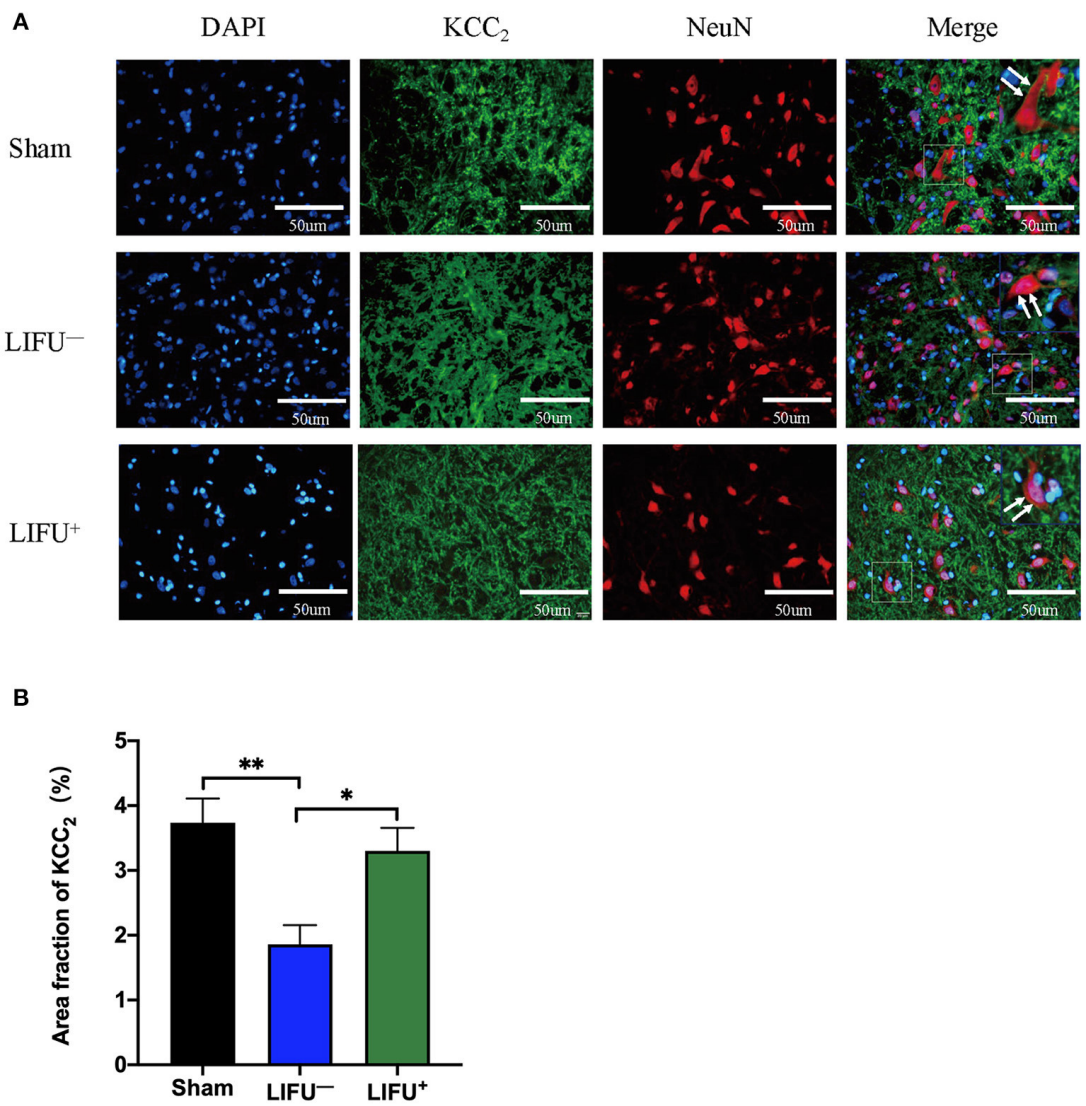
Immunofluorescence (IF) staining showing the expression of KCC. **(A)** Positive expressions of KCC_2_ are stained green and neurons are stained red; the expression of KCC_2_ was merged on the membranes of neurons (white arrow). **(B)** Histogram showing expression of the KCC_2_ protein for the different groups. The results show that the intensity of KCC_2_ expression was significantly reduced (**P < 0.01, compared with the sham group; one-way ANOVA with LSD test; *n* = 5, mean ± SEM); following LIFU treatment, the intensity of KCC_2_ expression significantly increased (*P < 0.05, compared with the LIFU^−^ group; one-way ANOVA with LSD test; *n* = 5, mean ± SEM). Scale bar = 50 μm.

Immunofluorescence was used to observe the effect of LIFU on the changes in KCC expression in lumbar spinal cord. The results showed that KCC_2_ had co-expression with the neurons, as shown in Figure 6A. The ImageJ software analysis showed that expression of KCC_2_ was reduced in the LIFU^−^ group compared with the sham group (*P* < 0.05), while expression of KCC_2_ increased in the LIFU^+^ group compared with those in the LIFU^−^ group (*P* < 0.05), as shown in Figure 6B.

## Discussion

Most studies have reported that neuromodulation techniques have been extensively studied and proved to be effetely in many diseases' treatment (Pan et al., 2022; Wang, 2022), such as repetitive functional magnetic stimulation (rFMS) alleviated the urinary retention for the patients after spinal cord injury (Zhang et al., 2022), promote the rehabilitation after brain tumor surgery (Dadario et al., 2022); Electrical nerve stimulation, which has been used for treating neuropathic pain, has been shown to be an effective treatment for reducing spasticity (Fernández-Tenorio et al., 2019; Tapia Pérez, 2019). The reduction in spasticity by functional electrical stimulation (FES) has also been confirmed by clinical studies, including a significant reduction in quadriceps tone in incomplete spinal cord patients and an increase in voluntary muscle strength after receiving FES (Granat et al., 1993). A randomized trial of different modalities of electrical stimulation demonstrated that FES significantly improved spasticity following SCI (Sivaramakrishnan et al., 2018). Physical rehabilitation training has also been used for treating spasticity. After receiving bike training, the spasticity of SCI rats was significantly reduced (Beverungen et al., 2020). A prospective clinical trial showed that passive rhythmic leg exercise significantly reduced spasticity in SCI veterans (Rayegani et al., 2011). Another study also confirmed that combined FES and passive leg movements successfully reduced spastic muscle tone in SCI patients (Krause et al., 2008). In this study, we first used ultrasound to stimulate the spinal cord in order to treat spasticity and found that LIFU stimulation significantly reduced spasticity in SCI rats.

LIFU stimulation has been regarded as a potential neuromodulatory method for treating many neurological disorders, and studies of LIFU stimulation for neuromodulation have moved from rodents to non-human primates to humans (King et al., 2013; Ai et al., 2018). King et al. (2013) applied LIFU to stimulate the brain and successfully induced a motor response. A recent study has also confirmed that LIFU with a higher duty cycle (DC = 70%) produced excitatory neuromodulatory effects, while a lower duty cycle (DC = 5%) produced suppressive neuromodulatory effects (Lee et al., 2016; Yoon et al., 2019; Kim et al., 2021). In a clinical study, transcranial ultrasound stimulation significantly upregulated the memory network of Alzheimer's patients (Beisteiner et al., 2020). In the present study, spasticity in rats following SCI was evaluated using the methods reported in the literature (Plantier et al., 2019), and our results confirmed that spasticity of the hind limbs in rats with SCI was significantly alleviated (Figure 3) and motor function significantly improved (Table 1) following LIFU (DC = 50%) stimulation of the spinal cord. Electrophysiological tests (FDD and EMG) also confirmed that LIFU stimulation successfully alleviated spasticity (Figure 4). The results suggest that LIFU stimulation of the spinal cord has a potentially important value in the treatment of spasticity following SCI.

A previous study showed that SCI leads to damage of the reticulo-spinal pathways, which reduces activation of Renshaw cells (Mazzocchio and Rossi, 1989, 1997). From previous studies, FES and exercise training were found to activate mainly the Renshaw cells in the spinal cord by stimulating the peripheral nerves, thus increasing negative feedback by the Renshaw cells to the α motor neurons and reducing spasticity (Aydin et al., 2005; Sivaramakrishnan et al., 2018). A study by Ahmed and Wieraszko (2012) showed that activation of the sensorimotor cortex by weak electrical signals significantly increased the expression of GABAergic spinal interneurons and GABAergic terminals, and also showed that stimulation of the spinal cord by electrical currents also increased the release of D-2,3-3H-aspartic acid, which restored motor control for SCI patients (Ahmed and Wieraszko, 2012). In animal experiments, it has been found that LIFU can cause behavioral and electrophysiological changes when the animals received central nervous system regulation (Tufail et al., 2010; Cheng et al., 2014). In previous studies, we confirmed that LIFU can activate the neurocircuits of the spinal cord effectively (Liao et al., 2021a). Li et al. (2020) applied ultrasound-driven piezoelectric current to successfully activate the spinal cord neurocircuits of SCI rats. A clinical study confirmed that epidural electrical stimulation can modulate the spinal network of complete SCI patients and help recovery of the motor function of paraplegic patients (Gill et al., 2018; Ridler, 2018). Thus, damaged spinal cords can also be activated and modulated. The present study also successfully activated the spinal cord neurocircuits with LIFU stimulation, recorded by EMG in the sham and SCI groups (Figure 2). Thus, we speculated that LIFU activation of spinal cord nerve circulation may increase the inhibitory effect of spinal cord interneurons, and thus reduce spasticity following SCI.

**Figure 7:**
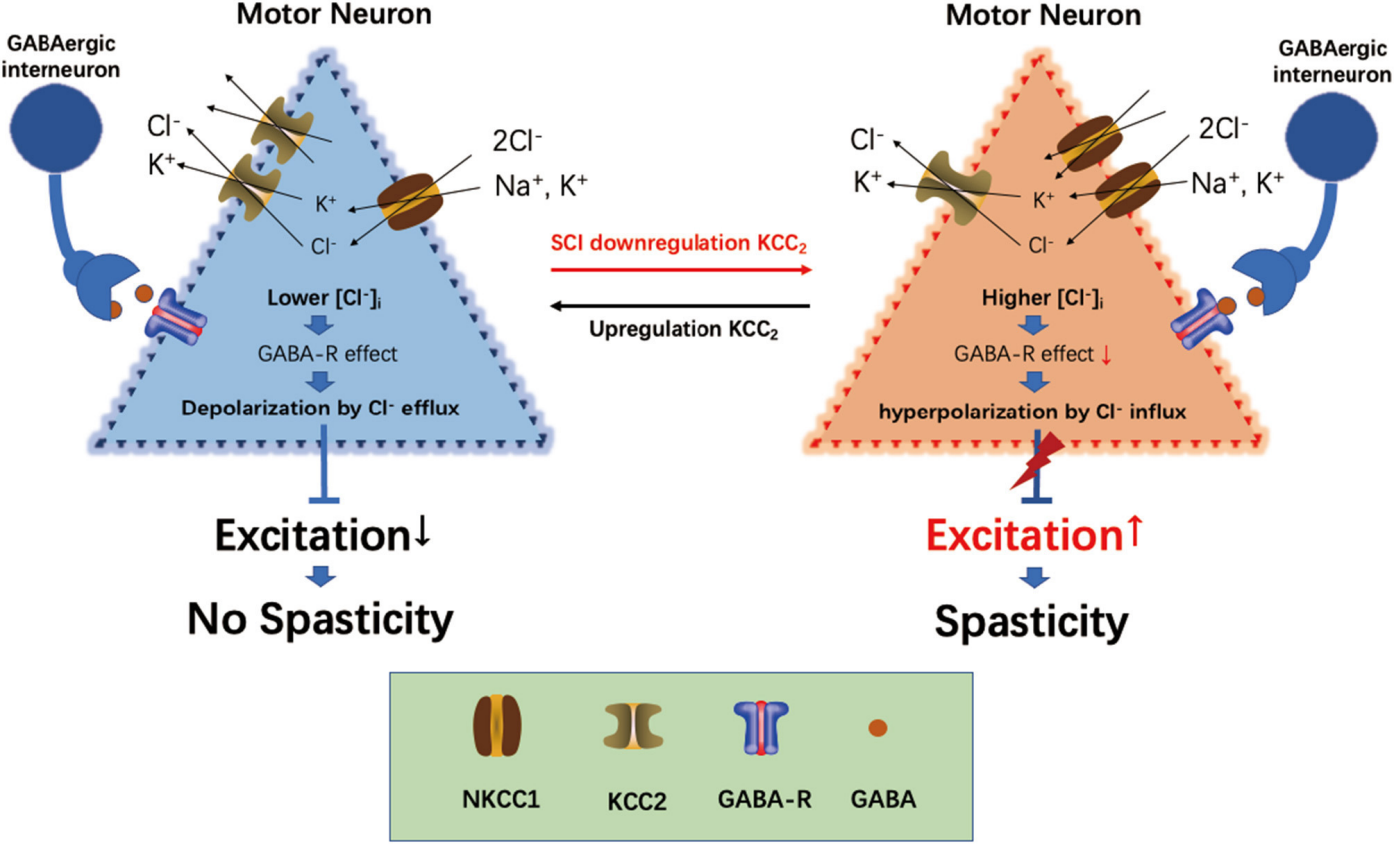
Overview of current views on the role of KCC_2_, NKCC1, and GABA in spasticity induction following spinal cord injury (SCI).

GABA is the main inhibitory neurotransmitter present in the central nervous system, and its inhibitory effect depends on its achieving a lower gradient of [Cl^−^]_i_ in cells (Kahle et al., 2008; Ben-Ari et al., 2012). The intracellular [Cl^−^]_i_ concentration gradient in neurons is established and maintained by the cation-chloride cotransporter (Slc12a) family on the neural membrane (Delpire and Mount, 2002; Payne et al., 2003). KCC_2_ (Slc12a5) is the only Cl^−^-extruding protein present in neuron cells, which extrude intracellular Cl^−^ to the extracellular environment to maintain a lower Cl^−^ concentration in neuron cells, thus maintaining the inhibitory effect of GABA (Boulenguez et al., 2010). The Na^+^-K^+^-2Cl^−^ cotransporter NKCC1 (Slc12a2) is the Cl^−^-importing protein, which transports extracellular Cl^−^ into cells, thus maintaining a higher intracellular [Cl^−^]_i_ concentration in neurons and reducing the inhibitory effect of GABA on neurons. The interaction between KCC_2_ and NKCC1 maintains Cl^−^ homeostasis in neurons and regulates the inhibitory effect of GABA (Plotkin et al., 1997; Delpire and Staley, 2014). Following SCI, the expressions of KCC_2_ and NKCC1 were mutually regulated through the with-no-lysine [K] (WNK) kinase pathway, and the protein level between downregulation of KCC_2_ and upregulation of NKCC1 showed a significant negative correlation (Côté et al., 2014; Kahle and Delpire, 2016). The downregulation of KCC_2_ and upregulation of NKCC1 in the lumbar spinal cord led to an increased intracellular concentration of [Cl^−^]_i_ in the neurons and destroyed homeostasis (Côté et al., 2014). The breaking of [Cl^−^]_i_ homeostasis also reduced the inhibitory effect of GABA and increased the sensitivity of the motor neurons (Viemari et al., 2011), which resulted in spasticity (Kahle et al., 2008; Côté et al., 2014) (Figure 7).

Following upregulation of KCC_2_ expression and downregulation of NKCC1 expression in the lumbar spinal cord by drugs or exercise treatment, hind limb spasticity was significantly reduced (Côté et al., 2014; Beverungen et al., 2020). In this study, we used IF staining to test the expression of KCC_2_, and the results showed that KCC_2_ was co-expressed on the neuron membrane and LIFU treatment significantly improved the expression of KCC_2_ (Figures 6A,B). WB also showed that the expression of KCC_2_ in the lumbar spinal cord of rats in the LIFU^−^ group was significantly downregulated compared to that in the sham group. In the LIFU treatment group, the expression of KCC_2_ in the lumbar spinal cord was upregulated (Figure 5), and hind limb spasticity was also reduced (Figure 3C). Based on these results, we speculated that LIFU stimulation also enhances the effectiveness of GABA by upregulating KCC_2_ expression, which then reduces spasticity.

Spasticity is a common complication of SCI, which seriously reduces patients' quality of life (Maynard et al., 1990; Westgren and Levi, 1998; Holtz et al., 2017). Clinically, the treatment of spasticity following SCI includes the use of oral baclofen, baclofen pump (Dykstra et al., 2007; de Sousa et al., 2022), botulinum toxin injection (Picelli et al., 2019), and motor nerve blocking (Demir et al., 2018; Zhang et al., 2021). These treatments have various side effects or require surgery. Therefore, it is important to find a specific physical rehabilitation method with limited side effects and overall positive outcomes. Previous studies have confirmed that LIFU can activate spinal cord neurocircuits effectively and not result in SCI. In this study, we also demonstrated that LIFU can activate spinal neurocircuits and decrease spasticity following SCI in rats. These results provide a theoretical basis for the clinical application of LIFU in the treatment of spasticity and a new and effective rehabilitation procedure for spasm patients following SCI.

## Conclusion

The results of our study suggest that low-intensity focused ultrasound (LIFU) stimulation can activate the spinal cord neurocircuits successfully and alleviate spasticity in rats effectively following spinal cord injury (SCI). Effective treatment may be related to up-expression of KCC_2_ in the cell membranes of neurons by LIFU stimulation.

## Data Availability Statement

The original contributions presented in the study are included in the article/Supplementary Material, further inquiries can be directed to the corresponding author/s.

## Ethics Statement

The animal study was reviewed and approved by Animal Ethics Committee of Kunming Medical University.

## Author Contributions

YL and L-JA contributed to the design of the study. Y-HL, K-XL, M-XC, and BW contributed to the acquisition of data. Y-HL, YL, and S-CC contributed to the statistical analysis. Y-HL and YL drafted the manuscript. YL, S-CC, and L-JA revised the manuscript. All authors contributed to the article and approved the submitted version.

## Funding

This work was supported by the National Natural Science Foundation of China (Grant Nos. 81960421 and 82060421), the Science and Technology Innovative Team Grant of Kunming Medical University (No. CXTD201905), and the Basic Research Program of Yunnan Science and Technology Department (No. 202101AT070255).

## Conflict of Interest

The authors declare that the research was conducted in the absence of any commercial or financial relationships that could be construed as a potential conflict of interest.

## Publisher's Note

All claims expressed in this article are solely those of the authors and do not necessarily represent those of their affiliated organizations, or those of the publisher, the editors and the reviewers. Any product that may be evaluated in this article, or claim that may be made by its manufacturer, is not guaranteed or endorsed by the publisher.

## Supplementary Material

Supplementary Video 1 This shows a normal hind limb motor movement response to tail pinching in the sham operation rats (related to Figure 3A) and spinal cord injury (SCI) rats (related to Figure 3B).

Supplementary Video 2 Electromyograph (EMG) showing a typical activation of the spinal cord neurocircuits by LIFU^+^ stimulation for the sham operation rats (related to the Figure 2 sham group), LIFU^−^ stimulation for the sham operation rats (related to the Figure 2 LIFU^−^ group), and LIFU^+^ stimulation in the spinal cord injury (SCI) rats (related to the Figure 2 LIFU^+^ group).
